# Efficacy of *Khaya grandifoliola* Stem Bark Ethanol Extract in the Treatment of Cerebral Malaria in *Swiss albino* Mice Using *Plasmodium berghei* NK65 Strain

**DOI:** 10.1155/2023/5700782

**Published:** 2023-11-09

**Authors:** Gamago Nkadeu Guy-Armand, Yamssi Cedric, Noumedem Anangmo Christelle Nadia, Tako Djimefo Alex Kevin, Tientcheu Noutong Jemimah Sandra, Ngouyamsa Nsapkain Aboubakar Sidiki, Mounvera Abdel Azizi, Vincent Khan Payne

**Affiliations:** ^1^Department of Animal Biology, Faculty of Science, University of Dschang, P.O. Box 067, Dschang, Cameroon; ^2^Department of Biomedical Sciences, Faculty of Health Sciences, University of Bamenda, P.O. Box 39, Bambili, Cameroon; ^3^Department of Microbiology, Hematology and Immunology Faculty of Medicine and Pharmaceutical Sciences, University of Dschang, P.O. Box 96, Dschang, Cameroon; ^4^Department of Animal Organisms, Faculty of Science, University of Douala, P.O. Box 24157, Douala, Cameroon

## Abstract

**Background:**

Cerebral malaria is one of the most severe and dangerous forms of malaria and is potentially fatal. This study was aimed at evaluating the anticerebral malaria efficacy of *Khaya grandifoliola* used by traditional healers.

**Method:**

Fifty grams of *Khaya grandifoliola* stem bark was macerated in 1 L ethanol (95%) for 72 h. The filtrate was dried at 40°C until the obtention of a dry extract. The antimalarial test was evaluated using the Peter 4-day suppressive test and the Rane curative test. Mice were group into 6 groups of 6 mice each. For the antioxidant test, parameters such as malondialdehyde (MDA), superoxide dismutase (SOD), glutathione (GSH), catalase (CAT), and nitric oxide (NO) were assessed. The livers of mice were crushed and centrifuged in order to be measured. Aspartate aminotransferase (ASAT) and alanine aminotransferase (ALAT) using the Dutch Diagnostics Kit and blood were collected for haematological parameters.

**Results:**

The ethanol extract showed a suppressive activity of 78.12%, 75.30%, and 68.69% at 500 mg/kg, 250 mg/kg, and 125 mg/kg, respectively. Similarly, the curative activity showed a statistically significant reduction in parasitemia (*p* < 0.05). Antioxidant parameter assays showed a low value of MDA and a high value of SOD, CAT, NO, and GSH in the negative control group. A statistically significant higher values of ASAT and ALAT were observed in the negative control compared to the other test groups (*p* < 0.05). Haematological parameters showed a statistically significant decrease in white blood cells, red blood cells, haemoglobin, and platelets in the negative control group (*p* < 0.05).

**Conclusion:**

The results of this study justify the traditional usage of *Khaya grandifoliola* in the treatment of cerebral malaria. However, *in vivo* toxicity assessment is still necessary to verify its safeness.

## 1. Introduction

Malaria is one of the world's most important parasitic endemic diseases, and about 3.2 billion people are at risk of being infected worldwide. The most affected regions are Africa, Asia, Latin America, and the Middle East [1, 2]. In 2019, the World Health Organization (WHO) estimated that malaria is responsible for approximately 241 million cases and 627 thousand deaths worldwide [3]. Cerebral malaria is of public health concern in Cameroon. It is the third most affected country in Central Africa with 2.9% of malaria cases and 2.4% of deaths worldwide [4].

According to Nadia et al. [5], during a malaria attack, malaria parasites could create oxidative cascades in an organism that may lead to the production of toxic free heme, resulting in excessive production of free radicals, the main source of oxidative stress. The use of an antioxidant as a supplement or the use of a plant with the ability to inhibit both oxidative stress and various stages of plasmodium may be a better strategy for malaria control. In addition to this high and increasing prevalence, there is also low accessibility to healthcare facilities due to the relatively high cost of the drug and the unavailability of the drug in some areas [6].

The emergence of resistance of plasmodium strains, combined with the resistance of the vector to insecticides, is a problem to the control of malaria [7, 8]. One of the solutions to this problem of resistance is the usage of medicinal plants. Medicinal plants are accessible and affordable to the local population. In some African countries, such as Mali and Guinea, the prevalence of malaria has been reduced, thanks to the use of traditional medicines [9]. In Nigeria, work carried out on the chloroquine-resistant *P. falciparum* clone W2 showed that the aqueous extract of *K. grandifoliola* was active, with an IC_50_ of 15.2 *μ*g/mL [10]. In the Western Region of Cameroon, *K. grandifoliola* is used for the treatment of cerebral malaria by traditional healers. Guy-Armand et al. [11] have demonstrated antiplasmodial, antioxidant, and cytotoxicity activities of ethanol and aqueous extracts of *K. grandifoliola* stem bark. The aqueous and ethanol extracts of *K. grandifoliola* bark had IC_50_ values of 54.27 ± 2.41 *μ*g/mL and 31.19 ± 4.06 *μ*g/mL, respectively, for the chloroquine-resistant PfDd2 strain and 53.06 *μ*g/mL and 28.03 ± 1.90 *μ*g/mL for the chloroquine-sensitive Pf3D7 strain. These results indicate a promising activity of the ethanol extract compared to the aqueous extract according to the classification of Kumari et al. [12].

However, according to Nadia et al. [5], extract may be active *in vitro* and inactive *in vivo* due to phenomena such as bioavailability, absorption, and biotransformation. It is therefore of paramount importance to evaluate the *in vivo* antimalarial and antioxidant activities of the ethanol extract of *K. grandifoliola*. This study was aimed at evaluating the antimalarial activity of *K. grandifoliola* stem bark ethanol extract, in order to justify its traditional usage.

## 2. Material and Methods

### 2.1. Collection and Identification of Plants

The leaves, flowers, fruits (used for identification), and stem bark (used for preparation of extracts) of *K*. *grandifoliola* were collected in June 2021 in Foumbot subdivision, West Region of Cameroon. The identification was done at the National Herbarium and registered under the code 52658/HNC.

### 2.2. Preparation of the Ethanol Extract

The method described by Guy-Armand et al. [11] was used for the preparation of the ethanol extract. Briefly, 10 *μ*L of 1% DMSO was first added to 50 g of the stem bark powdered, was mixed with 500 mL of ethanol (95%), and was homogenized for 72 h. The filtrate obtained was filtered (Whatman paper No. 1) and dried in an oven at 40°C until the obtention of a dry powder.

### 2.3. Parasite

The strain of *Plasmodium berghei* (NK65) was obtained from BEI Resources (Manassas, VA, USA) and maintained by subpassage in laboratory mice.

### 2.4. Antimalarial Activity

#### 2.4.1. The Peter 4-Day Suppression Test

The suppressive activity was carried out as described by Peters et al. [13]. *Swiss albino* mice of both sexes having a mean weight of 24 ± 2 g were used for the test. Thirty (30) mice out of 36 were infected each with 200 *μ*L of diluted inoculum containing 1 × 10^7^ parasitized erythrocytes intraperitoneally and then randomly divided into 5 groups of six (6) mice each. The remaining six nonparasitized mice formed the normal group as shown below:

- Group 1: infected and treatment with 500 mg/kg (*n* = 6)
- Group 2: infected and treatment with 250 mg/kg (*n* = 6)
- Group 3: infected and treatment with 125 mg/kg (*n* = 6)
- Group 4: infected and treatment with chloroquine (5 mg/kg) (*n* = 6)
- Group 5: infected and treatment with 1% dimethyl sulfoxide (DMSO) (*n* = 6)
- Group 6: normal control group (*n* = 6)

Mice were inoculated intraperitoneally with parasitized erythrocytes below the diaphragm as described by Azizi et al. [14]. Three hours (3 h) after inoculation, the mice were treated for 4 consecutive days (day 0, day 1, day 2, and day 3). At intervals of 24 h, on the 4th to 9th days, thin smears were prepared from the tail blood of each mouse and stained with the May-Grünwald-Giemsa. The parasitemia, average suppression of parasitemia, and mean survival rate were calculated as follows [14]:

(1) $$\begin{array}{c} \%\text{parasitemia}=\frac{\text{number of parasitized red blood cells}}{\text{total number of red blood cells count}}*100,\\ \%\text{suppression}=\frac{\text{average parasitemia of the negative control}-\text{average parasitemia of tested group}}{\text{average parasitemia in negative control}}*100. \end{array}$$

The mean survival rate of each group was determined over 30 days (D0-D29).

(2) $$\begin{array}{c} \text{Mean survival rate}=\frac{\text{number of survival day}}{\text{total number of }30\text{ days }}. \end{array}$$

#### 2.4.2. The Curative Test

The curative test was performed according to the modified method of Ryley and Peters [15]. Thirty-six *Swiss albino* mice of both sexes with a mean weight of 25 ± 2 g were used. Thirty (30) of 36 mice received 200 *μ*L of inoculum containing 1 × 10^7^ parasitized erythrocytes intraperitoneally on the first day of the test (D0). These mice were randomly divided into five groups. The remaining six (6) nonparasitized mice formed the normal group (group 6) as in the suppressive test above. Seventy-two hours (72 h) after inoculation, extracts at different doses were administered to the mice for 4 days (D3-D6). The parasitemia in all experimental groups was evaluated from the 4th to 10th (D3-D9) days. The parasitemia and mean survival rate was calculated.

(3) $$\begin{array}{c} \%\text{reduction}=\frac{\text{average parasitemia of negative control}-\text{average of parasitemia of tested group}}{\text{average of parasitemia of negative control}}*100. \end{array}$$

*(1) Tissue Preparation for Enzyme Assays*. On day 10 of the curative test, three mice per group were randomly selected and sacrificed. Blood was drawn from dry tubes for ASAT and ALAT determination and from heparinized tubes for evaluation of haematological parameters. Enzymatic stress parameters (SOD, CAT) and nonenzymatic stress parameters (MDA, glutathione, and NO) were measured. The liver was also sampled, weighed, and ground individually in a mortar.

*(2) Enzyme Assays*. Catalase activity was determined using the method of Tseuguem et al. [16] in which 50 *μ*L of tissue homogenate, 500 *μ*L of phosphate buffer (0.1 M, pH = 7), and 200 *μ*L of H_2_O_2_ (0.2 M) were introduced into test tubes. One minute later, 1 mL of potassium dichromate (5%) was added to the mixture. After incubation in a boiling water bath, the disappearance of the H_2_O_2_ substrate was measured spectrophotometrically at 570 nm.

Total SOD activity was assessed by the method of Şehirli et al. [17], using a 3 mM (instead of 0.3 mM) adrenaline solution prepared in a pH = 4 medium. In cuvettes, 70 *μ*L of tissue homogenate, 830 *μ*L of carbonate buffer (pH = 10.2), and 100 *μ*L of adrenaline (3 mM) freshly prepared in Tris buffer (pH = 4) were introduced. The absorbance of the adrenochrome was read at 480 nm, at 60 and 120 seconds after initiation of the reaction, against a blank.

GPx activity was determined using the method described by Şehirli et al. [17], based on NADPH oxidation. 50 *μ*L of tissue homogenate was added to 200 *μ*L of Tris buffer (10 mM, pH = 7.4), 1 mL of sodium hydrogen phosphate dihydrate (Na_2_HPO_4_, 2H_2_O; 0.3 M), and 0.1 mL of dithiobisnitrobenzoate (0.4 mg/mL in 1% trisodium acid). Absorbance was determined by spectrophotometer at 340 nm.

*(3) Nonenzymatic Assay*. Lipid peroxidation was measured using thiobarbituric acid (TBA) to form a pink chromogen that can be measured spectrophotometrically. Liver NO concentration was determined by the Griess reaction according to Tchatat Tali et al. [18]. The reaction mixture consisted of reduced nicotinamide adenine dinucleotide phosphate (NADPH), flavin adenine dinucleotide, and nitrate reductase, and the absorbance was measured spectrometrically at 540 nm.

*(4) Measurement of Transaminases*. Liver function indicators (ALAT and ASAT) were assessed using the Dutch Diagnostics Kit.

### 2.5. Ethical Consideration

All authors hereby declare that the “Principles for the Care of Laboratory Animals” (NIH Publication No. 85-23, revised 1985) have been followed, as well as specific national laws, where applicable [19]. All experiments were reviewed and approved by the Department of Animal Biology, Faculty of Sciences, University of Dschang.

### 2.6. Statistical Analysis

The data were entered into Microsoft Excel version 16.0 to calculate the percentages of inhibition and reduction of parasitemia and then transferred to GraphPad version 8.4 to plot the reduction curves and for the calculation of the survival means with ANOVA test.

## 3. Results

### 3.1. Suppressive Activity and Mean Survival Rate

Table 1 shows the suppressive effect and the mean survival rate of the ethanol extract. From this table, the doses 500 mg/kg, 250 mg/kg, and 125 mg/kg significantly inhibited the development of the parasite (*p* < 0.05) compared to the group receiving 1% DMSO (*p* < 0.05). As for the mean survival rate, the group receiving 1% DMSO recorded the lowest mean survival compared to the groups receiving chloroquine and the groups treated with different doses of ethanol extract. This difference was statistically significant (*p* < 0.05).

### 3.2. Curative Activity of the Ethanol Extract of *K. grandifoliola*

**Figure 1:**
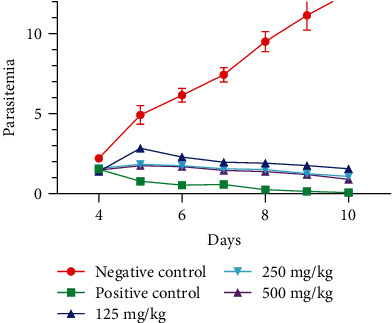
Evolution of parasitemia with respect to days.

Figure 1 shows the evolution of parasitemia with respect to day. A statistically significant difference between the parasitemia at different doses (500 mg/kg, 250 mg/kg, and 125 mg/kg) of the ethanol extract (*p* > 0.05) was observed. Similarly, the overall comparison of treatments showed significantly lower parasitemia levels in the treated groups (*p* < 0.05) compared to the 1% DMSO treatment.

#### 3.2.1. Effect of Ethanol Extract on the Mean Survival Rate

Table 2 presents the effect of the ethanol extract on the mean survival rate. It can be seen that the groups receiving chloroquine and ethanol extract treatments had significantly higher mean survival rates than the group receiving the 1% DMSO treatment. This difference is statistically significant (*p* < 0.05).

#### 3.2.2. Antioxidant Activity

*(1) Nonenzymatic Antioxidant Parameters*.  Table 3 shows the effect of ethanol extract of *K. grandifoliola* on nonenzymatic antioxidant parameters. It can be seen that the group treated with 1% DMSO had the highest values for MDA and NO, respectively, compared to the values obtained in the other test groups. This difference was statistically significant (*p* < 0.05). As for the GSH assay, the group treated with 1% DMSO had the lowest value compared to the other test groups. This difference was also statistically significant (*p* < 0.05).

*(2) Enzymatic Antioxidant Parameters*.  Table 4 shows the effect of the ethanol extract on enzymatic antioxidant parameters. It can be seen that the group treated with 1% DMSO had the lower value. This difference was statistically significant (*p* < 0.05).

#### 3.2.3. Biochemical Parameters

Table 5 shows the effect of ethanol extract of *K. grandifoliola* on biochemical parameters. Aspartate aminotransferase and alanine aminotransferase levels were higher in the 1% DMSO-treated group than in the other groups. This difference was statistically significant (*p* > 0.05). As for protein, the group treated with 1% DMSO had the highest level compared to the other groups. This difference was statistically significant (*p* < 0.05).

#### 3.2.4. Effects of Ethanol Extracts on Haematological Parameters

Table 6 shows the effect of the ethanol extract on blood parameters. In relation to white blood cells, the negative control group had the lowest mean (2.55 ± 0.53^b^) compared to the other groups. This difference was statistically significant (*p* < 0.05). Similarly, the mean assessment of monocytes also revealed a statistically significant difference (*p* < 0.05). As for granulocytes, blood platelets, red blood cells, and haemoglobin levels, their evaluation in the different treatment groups shows a statistically significant difference (*p* < 0.05).

## 4. Discussion

The suppressive activity of ethanol extract of *K. grandifoliola* bark on *P. berghei* development at different doses showed a high percentage of chemosuppression. These percentages of chemosuppression were dose-dependent with 78.12%, 75.30%, and 68.69% at doses 500 mg/kg, 250 mg/kg, and 125 mg/kg, respectively. From these percentages, we note that the extract is considered to have a good antiplasmodic activity according to the classification of Muńoz et al. [20] which stipulates that an extract has a good activity when, at a dose of 250 mg/kg/day, it leads to a decrease greater than or equal to 50% of the parasitemia. Similar observations were also done by Gouissi et al. [21] in Cameroon with the extract of *K. grandifoliola* which also presented a high percentage of dose-dependent chemosuppression with 71.80% and 59.42%, respectively, at doses 150 mg/kg and 300 mg/kg. The work of Agbedahunsi et al. [22] and Makinde et al. [23] conducted in Nigeria on the stem bark of *K. grandifoliola* with organic (*n-hexane*, chloroform, and ethyl acetate) and nonorganic (distilled water) solvent extracts, respectively, obtained a high percentage of dose-dependent chemosuppression. In contrast, Agbedahunsi et al. [22] presented low chemosuppression percentages of methanol extract 59.9, 47.8, and 45.5 at 400, 200, and 100 mg/kg, respectively. The curative activity of the ethanol extract of *K. grandifoliola*, at doses 500, 250, and 125 mg/kg, showed a dose-dependent and statistically significant reduction in parasitemia compared to the negative control and positive control groups. This observation was also made by Adeyemo-Salami et al. [10] on the antioxidant and antiplasmodial activities of *Paullinia pinnata* methanol leaf extract and by Nadia et al. [5] on the antimalarial activity of ethyl acetate extract and *Bidens pilosa* fraction against *Plasmodium berghei*. The efficacy of *K. grandifoliola* in the treatment of cerebral malaria could be explained by phytochemical composition, alkaloids, terpenoids, tannins, saponins, and anthraquinons which are known to have antiplasmodial activity [24, 25] but also due to the nature of the solvent used to prepare the extract [26]. The suppressive activity of the *K. grandifoliola* extract is thought to be due to the accumulation of these bioactive compounds during the 3 days of treatment in the animals' blood, in particular, the alkaloids, which inhibit parasite protein synthesis, and the flavonoids, which chelate with the nucleic acid-base pair of the parasite, thus inhibiting parasite multiplication [27, 28]. The reduction of the parasite during the curative test in the treated groups could be justified by the presence of terpenoids, in particular, deacytlkhivorin in *K. grandifoliola*, which acts on all stages of Plasmodium development [27, 28].

Regarding the mortality rate, the negative control group, treated with 1% DMSO in the suppressive and curative test, had a high and statistically significant mean compared to the other test groups. This observation was also made by Bansal and Kaushal [29] when evaluating the antiplasmodial activity of *Nauclea latifolia* and by Muganga et al. [30] on the evaluation of the *in vitro* and *in vivo* antiplasmodial activity of three Rwandan medicinal plants. The low average survival rates observed in the negative control during these two tests could be explained not only by the fact that dimethyl sulfoxide (1% DMSO), unlike the ethanol extract of *K. grandifoliola* and chloroquine, does not possess properties capable of reducing and inhibiting the development of *P. berghei NK65*.

The presence of malaria parasites in the body initiates several cascade reactions that can lead to the production of several free radicals which are the main source oxidative stress [5]. The use of an antioxidant as a supplement to drug with the ability to inhibit both oxidative stress and different stages of Plasmodium may be an advantage.

The ability of ethanol extract of *K. grandifoliola* bark to reduce oxidative stress was evaluated by assaying parameters such as MDA, SOD, GSH, catalase, and NO which are parameters present in plasma that act as antioxidants by converting reactive oxygen species and reactive nitrogen species into stable compounds and by participating in the scavenging of excessive free radicals [31]. A higher level of MDA in the negative control group compared to the other treated groups was observed. This result is similar to the observations made by Ma et al. [32] on the *in vivo* antioxidant activity of deacetyl asperulosic acid in Sandrine et al. [33] when evaluating the cytoprotective and antioxidant properties of the aqueous extract of *Khaya grandifoliola* stem bark (Meliaceae) in rats. These low levels of MDA in the test groups treated with ethanol extract could reflect low liver damage in contrast to the negative control group [34, 35] and could be explained by the capacity of the extract to limit the oxidation of polyunsaturated fatty acids.

Superoxide dismutase and catalase were found to be lower in the negative control group than in the other test groups. This observation was also made by Onoja et al. [36] when evaluating the *in vitro* and *in vivo* antioxidant potential of *Aframomum melegueta* seed methanol extract and those of Gupta et al. [31] on the *in vivo* antioxidant activity of a topical cream based on *Cassia tora* leaf extract. Catalase is an enzyme present in all cells of the body that catalyses the decomposition of hydrogen peroxide, a reactive oxygen species, which is a toxic product of normal aerobic metabolism and of the pathogenic production of reactive oxygen species (ROS) [36], while SOD catalyses the dismutation of superoxide to hydrogen peroxide and oxygen, thereby reducing the likelihood of the superoxide anion reacting with nitric oxide to form reactive peroxynitrite [37]. The high level of catalase and SOD observed in this study could be due not only to the fact that the extract is effective against ROS and hence its antioxidant activity but also affects the immune system. Glutathione levels were lower in the negative control group than in the other test groups. The major role of glutathione is to eliminate lipid peroxides resulting from the action of oxidative stress on polyunsaturated fatty acids [38]. The high levels observed in the groups treated with ethanol extract reflect a good fight against the damage caused by oxidative stress induced in this case by the proliferation of *Plasmodium berghei* [39, 40]. Nitric oxide levels were higher in the negative control group than in the other tested groups. Nitric oxide could in some cases act as a scavenger of superoxide anion [41]. Its high value in the negative control group also reflects a high production of superoxide anion as a result of the various oxidative processes in the animal. When NO production is depleted, the superoxide anion can produce numerous free radicals as a result of the Haber-Weiss reaction [42]. These low NO values in the other test groups could reflect good management of oxidative stress by the ethanol extract.

The liver is the main organ indicating an alteration of some hepatic metabolic functions causing changes in ASAT and ALAT [18]. The results observed during this test showed a statistically significant increase of ASAT and ALAT in the untreated group compared to the other test groups (*p* ≤ 0.05). This observation was not only made by Nadia et al. [5] when testing the antimalarial activity of ethyl acetate extract and *Bidens pilosa* fraction against *P. berghei* but also by Tchatat Tali et al. [18] on the *in vivo* antiplasmodial activity of the aqueous extract of *Terminalia mantaly* bark in mice infected with *P. berghei*. They justified this by the ability of *Terminalia mantaly* extract to induce a decrease in enzymatic activities, underlining its protective effect on liver damage caused by *P. berghei*. In contrast, Chaniad et al. [43] did not show a statistically significant difference between the treated and untreated groups. The hyperproduction of the enzymes ASAT and ALAT observed in the negative control group compared to the other test groups during our work could be due to a great destruction of hepatocytes caused by hyperparasitemia. A higher level of protein in the negative control group compared to the other groups was observed. Proteins are organic macromolecules widely distributed in the body. They are divided into two fractions, globulins and albumins produced by the liver, whose role is also to monitor liver function [44]. The high protein level observed in the negative control group would reflect a high albumin level as the blood count showed a decrease in white blood cells and monocytes.

White blood cells and platelets play a role in immune defence against foreign bodies [5]. In this study, there was a lower white blood cell and platelet count in the 1% DMSO group compared to the other test groups (*p* < 0.05). On the other hand, Tchatat Tali et al. [18] when evaluating the *in vivo* antiplasmodial activity of the aqueous extract of *Terminalia mantaly* stem bark in mice infected with *P. berghei* as well as Nadia et al. [5] justified this higher white blood cell and platelet count by the fact that, unlike the extract and chloroquine, 1% DMSO does not have antiplasmodial activity, and therefore, the organism produces more antibodies to defend itself. The low rate of white blood cells and platelets observed during this study can be explained by the fact that on the 10th day of the test as opposed to the 5th day as observed in the studies cited above, the inflammatory reaction caused by the trophozoites is essential and this creates an increase in cell adhesion as well as an increase in the passage of white blood cells and platelets into the extravascular environment, thus causing a decrease in their rate in the blood. As for the red blood cell and haemoglobin levels, low levels were observed in the negative control group compared to the other test groups (*p* ≤ 0.05). This observation was made by Tchatat Tali et al. [18] when evaluating the *in vivo* antiplasmodial activity of the aqueous extract of *Terminalia mantaly* bark in mice infected with *P. berghei* as well as Jassim [45] when evaluating haematological and biochemical changes in albino rats receiving an aqueous extract of *Ocimum basilicum* leaves. The low RBCs and haemoglobin levels observed in the negative control group could be explained not only by the ability of the ethanol extract of *K. grandifoliola* stem bark to reduce and inhibit the development of *P. berghei* responsible for the destruction of erythrocytes during malaria episodes but also by the nontoxic effect on erythrocytes of the ethanol extract of *K. grandifoliola* and chloroquine on red blood cells [11, 46].

## 5. Conclusion

The ethanol extract of *Khaya grandifoliola* stem bark showed good antiplasmodial activity as well as reducing oxidative stress created by the proliferation of *P. berghei*. This study suggests that *K. grandifoliola* can be used in traditional medicine for the treatment of malaria. Although it has low *in vitro* cytotoxicity, evaluation of its *in vivo* toxicity is necessary to determine its safeness.

## Figures and Tables

**Table 1:** The suppressive effect and the mean survival rate of the ethanol extract on the development of *Plasmodium berghei*.

Treatment	Mean parasitemia	Chemosuppression	Mean survival
1% DMSO	83.25 ± 0.18^a^	NA	8.22 ± 1.62^a^
CQ 5 mg/kg	2.32 ± 0.009^b^	97.20	30.0 ± 0.00^b^
500 mg/kg	18.21 ± 0.03^c^	78.12	27.44 ± 2.226^c^
250 mg/kg	20.56 ± 0.06^c^	75.30	25.11 ± 3.234^c^
125 mg/kg	26.06 ± 0.01^d^	68.69	25.89 ± 2.751^c^

Values carrying the same superscript letter are not significantly different (*p* < 0.05). NA: not applicable.

**Table 2:** Effect of the ethanol extract on the mean survival rate.

Treatment	Mean survival
1% DMSO	8.22 ± 1.62^a^
CQ 5 mg/kg	30 ± 000^b^
500 mg/kg	26.89 ± 2.65^c^
250 mg/kg	25.11 ± 2.45^c^
125 mg/kg	24.89 ± 2.56^c^

Values carrying the same superscript letter are not significantly different (*p* < 0.05).

**Table 3:** Effect of ethanol extract of *K. grandifoliola* on nonenzymatic antioxidant parameters.

Treatment	GSH	MDA	NO
1% DMSO	11.29 ± 1.00^a^	2.029 ± 0.048^a^	26.26 ± 3.261^a^
CQ 5 mg/kg	29.92 ± 1.229^b^	0.95 ± 0.24^b^	8.954 ± 1.045^b^
Distilled water	17.05 ± 1.343^c^	0.1667 ± 0.24^c^	19.47 ± 4.307^c^
500 mg/kg	18.98 ± 5.378^c^	1.34 ± 0.164^b^	12.62 ± 4.852^c^
250 mg/kg	22.27 ± 4.18^c^	1.212 ± 0.167^b^	4.164 ± 1.27^d^
125 mg/kg	19.86 ± 0.99^c^	1.20 ± 0.2^b^	4.632 ± 1.354^d^

Values carrying the same superscript letter are not significantly different (*p* < 0.05).

**Table 4:** Effect of ethanol extract of *K. grandifoliola on* enzymatic antioxidant parameters.

Treatment	Superoxide dismutase	Catalase
1% DMSO	1.29 ± 0.4^a^	0.22 ± 0.02^b^
CQ 5 mg/kg	6.65 ± 0.82^b^	0.27 ± 0.03^a^
Distilled water	5.72 ± 0.45^b^	0.32 ± 0.03^a^
500 mg/kg	8.48 ± 0.73^b^	0.31 ± 0.03^a^
250 mg/kg	6.35 ± 0.37^b^	0.29 ± 0.02^a^
125 mg/kg	4.04 ± 0.45^c^	0.40 ± 0.05^c^

Values carrying the same superscript letter are not significantly different (*p* < 0.05).

**Table 5:** Effect of *K. grandifoliola* ethanol extract on biochemical.

Treatment	ASAT (serum)	ALAT (serum)	Protein (liver)
1% DMSO	88.61 ± 5.36^a^	55.04 ± 1.316^a^	178.7 ± 5.56^a^
Distilled water	64.31 ± 0.33^b^	22.70 ± 0.336^b^	66.16 ± 0.498^b^
CQ 5 mg/kg	63 ± 2.26^ab^	21.15 ± 0.89^b^	65.88 ± 2.95^b^
500 mg/kg	70.57 ± 3.99^c^	31.12 ± 2.21^c^	69.46 ± 2.698^b^
250 mg/kg	59.85 ± 2.69^d^	33.54 ± 1.37^c^	54.64 ± 3.791^c^
125 mg/kg	57.34 ± 1.97^d^	24.7 ± 1.93^b^	71.89 ± 1.64^d^

Values with the same letters (a, b, c, and d) are not statistically different at *p* < 0.05.

**Table 6:** Effect of ethanol extract on blood parameter.

Treatment	WBC	LYM	Mono	Gran	PLT	RBC	HGB	TMH	HTC	CCMH	MCV
500 mg/kg	5.807 ± 1.3^a^	48.50 ± 16.8^a^	11.4 ± 0.75^a^	16.40 ± 2.6^a^	733.3 ± 25.7^a^	5.8 ± 1.31^a^	9.76 ± 1.94^a^	20.10 ± 1.68^a^	28.83 ± 4.1^a^	40.53 ± 2.5^a^	47.8 ± 7.3^a^
250 mg/kg	5.190 ± 0.4^a^	46.13 ± 4.3^a^	10.43 ± 2.3^a^	14.20 ± 0.2^a^	582.3 ± 52.2^a^	5.2 ± 0.43^a^	11.60 ± 1.8^a^	18.63 ± 0.57^a^	19.93 ± 2.2^a^	39.47 ± 1.6^a^	44.53 ± 0.96^a^
125 mg/kg	4.997 ± 0.6^a^	67.87 ± 11.3^a^	11 ± 3.37^a^	17.23 ± 3.19^a^	589.3 ± 42.6^a^	5 ± 0.68^a^	10.4 ± 1.4^a^	20.90 ± 0.00^a^	20.30 ± 0.2^a^	35.4 ± 0.00^a^	45.4 ± 3.95^a^
CQ 5 mg/kg	6.303 ± 0.2^a^	65.63 ± 8.75^a^	13.13 ± 1.8^a^	11.57 ± 2.54^a^	560.3 ± 47.2^a^	6.3 ± 0.27^a^	9.43 ± 2.44^a^	18.97 ± 0.98^a^	38.37 ± 4.1^a^	37.6 ± 1.8^a^	47.57 ± 3.51^a^
1% DMSO	2.55 ± 0.53^b^	47.63 ± 5.83^a^	16.90 ± 3.9^b^	7.2 ± 1.623^b^	360.3 ± 35.7^b^	2.550 ± 0.5^b^	7.7 ± 2.1.8^b^	19.57 ± 1.86^a^	31.10 ± 3.0^a^	40.47 ± 1.2^a^	47.23 ± 3.90^a^
Normal	6.763 ± 0.3^a^	50.10 ± 6.45^a^	13.37 ± 4.15^a^	17.37 ± 9.73^a^	753.7 ± 57.9^a^	6.763 ± 0.3^a^	12.07 ± 1.5^a^	19.33 ± 0.92^a^	38.40 ± 4.1^a^	38.60 ± 0.6^a^	46.33 ± 5.35^a^

Values carrying the same superscript letter are not significantly different (*p* < 0.05). Values with the same letters (a and b) are not statistically different. WBC = white blood cells; LYM = lymphocytes; PLT = platelet; RBC = red blood cells; HGB = haemoglobin levels; Gran = granulocytes.

## Data Availability

All data generated and analysed are included in this research article.
